# Reducing maternal and child mortality in rural Ghana

**DOI:** 10.11604/pamj.2021.39.263.30593

**Published:** 2021-08-24

**Authors:** Joseph Adu, Shree Mulay, Mark Fordjour Owusu

**Affiliations:** 1 Faculty of Health Sciences, Department of Health and Rehabilitation Sciences, Western University, 1201 Western Rd, London, ON N6G 1H1, London, Ontario, Canada,; 2 Faculty of Medicine, Department of Community Health and Humanities, Memorial University of Newfoundland, 300 Prince Philip Dr, St. John's, NL A1B 3V6, St. John´s, Newfoundland, Canada,; 3 School of Health Sciences, University of Canterbury, Christchurch, New Zealand

**Keywords:** Maternal mortality ratio, infants’ mortality, rural Ghana, community-based health planning, services

## Abstract

The lack of health infrastructure in developing countries to provide women with modern obstetric care and universal access to maternal and child health services has largely contributed to the existing high maternal and infant deaths. Access to basic obstetric care for pregnant women and their unborn babies is a key to reducing maternal and infants´ deaths, especially at the community-level. This calls for the strengthening of primary health care systems in all developing countries, including Ghana. Financial access and utilization of maternal and child health care services need action at the community-level across rural Ghana to avoid preventable deaths. Financial access and usage of maternal and child health services in rural Ghana is poor. Lack of financial access is a strong barrier to the use of maternal and child health services, particularly in rural Ghana. The sustainability of the national health insurance scheme is vital in ensuring full access to care in remote communities.

## Commentary

### Introduction

Sub-Saharan Africa (SSA) accounted for the highest Maternal Mortality Ratio (MMR) in Africa, with 546 deaths/100,000 live births in 2015 compared to any other region in the world [1]. The MMR for SSA represented 65% of all maternal deaths in the developing world [1]. Improvements in maternal and infant mortality in developing countries over the years have not been uniform due to disparities in the distribution of human resources, infrastructural facilities, essential drugs, and quality of care [1]. Previous studies highlight poor quality of care, non-availability of care and cultural beliefs/ignorance of the need for obstetric care for these disparities [2, 3]. Many women do not want to deliver in institutions because of the poor quality of care and risk dying during perinatal period, especially in the rural areas of developing countries [2].

Ghana, a country in SSA made progress by reducing its MMR and Infant Mortality Ratio (IMR) from 740 to 319/100,000 live births and 80 to 41 infant deaths/ 1000 live births, respectively, between 1990 and 2015 (Ghana Statistical Service [4]. Ghana´s improvement in MMR and IMR was facilitated by rolling out innumerable policies by Ghana Health Services (GHS)/Ministry of Health (MOH) which brought about an increased usage of facility delivery and postnatal care [5]. These policies include the Ghanaian Government policies of exempting pregnant women from delivery fees in all public and religious health institutions since September 2003, followed by implementation of free maternal healthcare policy in July 2008, as well as the free maternal healthcare policy under the National Health Insurance Scheme (NHIS) [5]. While the improvements made are commendable, Ghana still needs to focus on increasing its rates of live births and universal access to maternal healthcare. According to the 2017 Ghana Maternal Health Survey (GMHS), maternal mortality in Ghana accounted for 14% of all deaths; 10% from direct maternal causes and 4% from indirect maternal causes, suggesting that two-thirds of deaths (67%) were direct maternal deaths [4]. In addition, more than one quarter (27%) were indirect maternal deaths, and another 6% were due to unspecified maternal causes [4]. The common causes of direct maternal deaths are obstetric haemorrhage (30%), followed by hypertensive disorders (14%), and sepsis (10%) which results from complications due to non-medical abortions. The management of both obstetric haemorrhage and pregnancy-induced hypertension requires services of nurses, midwives, and doctors who are scarce in rural-Ghana [4, 5].

Neonatal deaths result from poor infant outcomes which account for 71% of all infant deaths in Ghana according to the Ghana Statistical Services figures in 2015. The GHS estimate that a neonate dies every fifteen minutes in Ghana, with over 21,000 deaths occurring annually as confirmed by the Ghana News Agency in 2017. The GMHS (2017) also reported IMR of 37 deaths/1000 live births and under-five mortality ratio of 52 deaths/1000 live births; implying 1 in 27 children die before their first birthday, whereas 1 in 19 children die before age five [4]. The cause of these deaths included complications associated with preterm birth, difficult labour, inadequate access to quality healthcare, financial constraints faced by mothers, mismanagement of the NHIS, and poor nutrition amongst pregnant women [4]. The GHS attributes the country´s setbacks on the poor performance of maternal and child health department of the GHS and the inadequate number of trained and certified midwives in the country, particularly within rural communities, where midwives and nurses often refuse rural posting after training as reported by the Ghana News Agency in 2017. Clearly, the GHS/MOH have a good grasp of the challenges in providing quality care for women and infants in hard-to-reach areas of the country, however, there are no robust policies to address these problems. This paper explores policies implemented to improve access to maternal health services in rural Ghana and ways to strengthen the Ghanaian NHIS for better uptake of maternal health services in rural Ghana. As well, strategies to improve pregnancy and childbirth outcomes to further reduce the high rates of maternal and infant mortality in rural-Ghana will be suggested.

### Improving maternal and infant health in rural Ghana

The enhancement of women´s health in Ghana largely depends on the reproductive and child health policies of the MOH and GHS. Available policy documents on maternal and child healthcare services by MOH/GHS indicate that Ghana has adequate policies in place to provide the needed services to improve maternal and child healthcare as shown in the 2013 annual report of the GHS. Undeniably, the maternal and child health policy guidelines follow the recommendations made by the International Conference on Population and Development in Cairo in 1994. Key components of these policies focus on improving access to universal education, reducing rates of infant, child, and maternal mortality, and increasing access to reproductive and sexual health services as outlined in the GHS 2013 annual performance report of the Family Health Division in 2014. However, progress in achieving these goals has been limited due to inconsistent and insufficient human and financial capital as well as infrastructural constraints. Investments in these areas require urgent attention from central government and Civil Society Organizations (CSOs) to improve access to basic obstetrics to all pregnant women and newborns in Ghana. The GHS has failed to implement some of the policies outlined by the MOH due to lack of funding and human resources [5].

Nevertheless, the implementation of the Community-Based Health Planning and Services (CHPS) program by the MOH/GHS in 2000 to improve access to maternal and child health services at the community-level to achieve MDG targets were effective [4, 6]. The CHPS contributed to scaling-up universal access to healthcare, especially maternal and child healthcare services in rural-Ghana [5, 6]. The CHPS provided entry point access to the Ghanaian healthcare system at the community-level, where community health nurses with basic knowledge in obstetrics examined a pregnant woman, provided antenatal care, and assisted during labour or referred the woman to a more advanced health center for further management. In addition, women received free maternal healthcare through a policy issued by the NHIS that enabled them to access antenatal care (ANC), facility-based delivery (FBD), and postnatal care (PNC) services. The publicly insured program played a significant roles in improving maternal and infant outcomes, as indicated in 2007 by the increased attendance of ANC and PNC, as well as increased rates in FBD [5]. Prior to the inception of the NHIS in 2004, access to healthcare services for pregnant women was privatized, and attendance was based on having financial resources. Women and families who lived in poverty, could not to afford services in some public and private hospitals where there was a cost, including having an FBD. The free maternal health policy under the NHIS made it possible for all pregnant women to access free healthcare, including FBD and Caesarean delivery. For example, the findings of previous studies in Ghana indicate an increase in FBD from 50.1% in 2004 to 71.2% in 2009 compared to 46 % between 1998 and 2003 when the NHIS was not instituted [7]. This clearly indicates that, before the inception of NHIS in 2004, about 53% of pregnant women delivered at home without the help of skilled attendants.

Despite the success of the new policy under the NHIS, a 2017 analysis of 2014 data from MOH and GHS showed that 90.1 % of pregnant women had ANC and 54.7% had FBD, respectively [5]. Even with the progress made, it appears that 45.3% of pregnant women still delivered at home without the services of a skilled birth attendant. These women likely risk maternal death or post-delivery complications, which could increase the already high MMR in Ghana. It also calls for needs assessment at the community-level to ascertain why significant proportions of women deliver at home instead of health facilities. As Austin *et al*. [2] found, some women are reluctant to go outside of the home to deliver their babies because of previous negative experiences and perceived poor quality of care. Women clearly want the option of home delivery, but home delivery needs to be safe to avoid post-delivery complications. It is important for staff to identify women who are likely to have a high-risk pregnancy during ANC and encourage them to go to a district hospital for specialized care at the time of delivery. Policy guidelines, such as those available in Rwanda are good examples for Ghana to follow, where policymakers worked to ensure facility improvements in infrastructure and personnel, effective medicines, and information campaigns, good transport systems, restrictions in home delivery and encouraging facility delivery [8]. The implementation of the aforesaid policies helped Rwanda to increase its facility-based deliveries to 90% [8]. Offering women with the possibility of having a supervised home delivery, whether it is a personal choice or a cultural preference, needs to be addressed to close the wide gap between home delivery and FBD to reduce the high MMR and IMR in Ghana. That said, the zero maternal mortality policy of the Shai-Osudoku District Hospital at Dodowa in the Greater-Accra Region which has yielded amazing results over the past five years could be adopted by policymakers to reduce preventable deaths in other district hospitals. While the district hospital documented an annual delivery of more than 2,000 cases over the years, the hospital has recorded no maternal deaths as documented by the Ghana News Agency in 2015.

The MOH and GHS can continue to work to achieve targets in rural-maternal health by strengthening the CHPS with additional infrastructure such as improved housing to accommodate staff, and other logistical supplies such as consumables and basic instruments for midwives, community health nurses to use, and incentivize postings in rural-health centres. Another important facet of accessible healthcare service is the provision of good road networks and safe transportation. The absence of safe road networks and transportation, coupled with the long distances between the CHPS compounds, district hospitals, and the communities that they serve, makes it difficult for women to access a healthcare facility [9]. This could even affect the triage system within a district should a midwife or community health nurse at the community clinic identify women with high-risk pregnancies and refer them to hospital for further management.

Developing infrastructure in rural Ghana requires new road networks, safe transportation systems, and optimal living conditions to support the CHPS in its mandate to make healthcare accessible to all. Good social amenities are also necessary to attract and retain health workers in rural Ghana. Achieving these goals requires inter-sectoral collaboration of all major sectors of the economy to address components that reflect each of their respective portfolios. A sustainable NHIS that provides free access to healthcare is also needed to increase the uptake of maternal and child health services which could be achieved by making the Ghana National Health Insurance Authority (NHIA) more financially viable. The NHIA is a corporate body under the auspices of the Government of Ghana that implements, operates, and manages the NHIS. The inability of the NHIA to timely pay providers and the high rates of non-reimbursement of NHIS claims could cause the withdrawal of services by providers [5, 10], which would significantly impact service delivery. The issues associated with the NHIS could be improved upon by the NIHA to maintain its use by the people of Ghana; all activities of the NHIA should be monitored by independent bodies to oversee fiscal and operational accountability. Continuous health promotion activities in the form of maternal education should be provided by healthcare professionals periodically to inculcate maternal health-seeking behavior among women in communities noted for poor use of healthcare services. Healthcare professionals could advertise their services by partnering with local media houses/radio stations to promote the importance of maternal healthcare services in their communities. Maternal health education should also target men at the community-level, particularly rural-communities where ANC, FBD, and PNC attendance is low to ensure their full participation in activities around childbirth and the use of skilled attendants during labour and contraceptives to control future pregnancies.

## Conclusion

Access to healthcare, including obstetric services, is essential in preventing maternal and neonatal deaths, especially in rural Ghana. The ability to provide access to care depends on several sectors working in partnership to develop more healthcare infrastructure, human resources staffing, effective and safe road networks, and reliable transportation systems. Well-equipped district hospitals with emergency obstetrics and neonatal care centers are vital for referrals from CHPS facilities to prevent deaths and undue complications.
